# Antiretroviral therapy use and CD4 cell count among people who inject drugs living with HIV in southern Vietnam

**DOI:** 10.1186/s12879-026-13180-2

**Published:** 2026-07-02

**Authors:** Duong Cong Thanh, David Charles Boettiger, Abu Abdul-Quader, Hoang Thi Thanh Ha, Pham Hong Thang

**Affiliations:** 1 Division of Global HIV and Tuberculosis, US Centers for Disease Control and Prevention, Hanoi, Vietnam; 2 The Kirby Institute, University of New South Wales, Sydney, Australia; 3 The National Institute of Hygiene and Epidemiology, Hanoi, Vietnam

**Keywords:** People who inject drugs, Vietnam, HIV, Integrated behavioral and biologic survey, Antiretroviral therapy, CD4, 95:95:95

## Abstract

**Objectives:**

People who inject drugs (PWID) are central to the HIV epidemic in Vietnam, many delay HIV testing and antiretroviral therapy (ART) until advanced disease. We assessed prevalence and correlates of ART use and low CD4 counts among PWID with HIV.

**Setting:**

Analysis of the 2013 Integrated Behavioral and Biologic Survey in Vietnam.

**Methods:**

Eligible participants were men aged ≥ 18 years who reported recent injecting, were HIV positive, and consented to future viral load (VL) and CD4 testing. Undetectable VL was defined as < 50 copies/ml. Percentages and prevalences were adjusted for sampling method.

**Results:**

Of 287 PWID, 55% knew their status. Among these, 88% were on ART, and 82% had undetectable VL. ART use was positively associated with older age (> 40 vs. < 30 years, OR 4.9, 95% CI 1.2–20.5) and opioid substitution therapy (OR 12.7, 95% CI 1.1–141.7). It was negatively associated with living alone (OR 0.1, 95% CI 0.0–0.5), living with a partner versus relatives (OR 0.3, 95% CI 0.1–0.8), higher income (> VND 4.5 vs. < 2.5 million; OR 0.2, 95% CI 0.1–0.7), and recent rehabilitation (OR 0.2, 95% CI 0.1–0.8). Among those not on ART, 16% had CD4 < 200 cells/mm³; longer injecting duration was strongly associated (OR 6.3 for > 10 vs. < 5 years, 95% CI 1.5–27.3).

**Conclusions:**

Suboptimal HIV status awareness, ART uptake, and advanced immunosuppression among untreated PWID highlight the need for strengthened case finding and earlier engagement. Linking treatment, social support, and HIV services in closed settings could improve outcomes.

**Supplementary Information:**

The online version contains supplementary material available at https://doi.org/10.1186/s12879-026-13180-2.

## Background

People who inject drugs (PWID) have been a population that substantially contributes to the HIV epidemic in Vietnam. While the national HIV prevalence was around 0.4% in the last decade, prevalence among people who inject drugs (PWID) was approximately 10% in 2013 and 10% in 2023. It was estimated that about one third in 2013 and 10% in 2024 of new HIV infections in Vietnam were attributable to injecting drug uses. It was estimated that number of PWID was about 123,000 in 2024 [1–3]. 

PWID with HIV in Asia also tend to initiate antiretroviral therapy (ART) at an advanced stage of disease [4] which leads to a poor short-term prognosis even with appropriate care. Provincial estimates indicate that the proportion of PWID who have received an HIV test in the past 12 months was low – generally under 40% [2, 5]. This has important public health implications as behaviors leading to HIV transmission are common among PWID and are unlikely to be modified in those who do not know their HIV-positive status.

Understanding current levels of HIV care engagement among PWID living with HIV is essential for achieving the UNAIDS 95-95-95 targets. This study documented the prevalence of ART use and the factors associated with using ART among PWID living with HIV in two cities in Viet Nam. Additionally, we described the prevalence of low CD4 cell counts among those not using ART, who remain at high risk of morbidity, mortality, and ongoing HIV transmission, and its associated factors. While the data are historical, the findings provide valuable insights into barriers to HIV treatment access, informing ongoing and future interventions tailored to meet the need of this population in Vietnam.

## Methods

### Survey

The 2013 Integrated Behavioral and Biologic Survey (IBBS) was conducted among PWID in 8 provinces (Dien Bien, Yen Bai, Quang Ninh, Hanoi, Nghe An, Ho Chi Minh City, Can Tho, and An Giang) in Vietnam. Our study specifically focused on a subgroup of participants who reported being PWID living with HIV and the analysis was limited to data from Ho Chi Minh City and Can Tho, the only two provinces with sufficient resources to conduct HIV viral load (VL) and CD4 cell count testing. Two sampling strategies were used in IBBS 2013: time-location sampling (TLS) and respondent-driven samplings (RDS). For these two southern provinces, we utilized respondent-driven sampling (RDS) to enroll a representative, cross-sectional sample of the local PWID populations. RDS is designed to approximate probability sampling in hard-to-reach or marginalized populations and has been described previously [6]. It uses a limited number of well-networked and representative recruits to refer their known peers who in turn refer additional waves of known peers.

Since injecting drug use in Vietnam occurs almost exclusively among men, only male-PWID aged ≥ 18 years were recruited. Injecting drug use was defined as self-reported injection of illicit drugs at least once in the last month. Behavioral data and blood sample collection occurred at study centers within government health facilities. Trained interviewers conducted face-to-face interviews using structured questionnaires, which covered sexual and drug use behaviors, as well as exposure to HIV prevention and treatment services. Interviewers entered and saved participant responses into computerized, handheld devices (tablets) from which data were downloaded and used to compile the final dataset. An English translation of the questionnaire is provided as a supplementary file (Additional file 1). Blood samples were collected following HIV pre-test counselling. Each participant was given an appointment card to receive their HIV test results two weeks after the interview.

### Laboratory

Plasma was tested for HIV antibody by enzyme-linked immunosorbent assay (Genscreen Ultra HIV Ag/Ab; Biorad, USA) and then confirmed by a second enzyme-linked immunosorbent assay (Murex HIV Ag/Ab Combination; DiaSorin, UK) and a rapid test (Determine HIV-1/2; Alere, Japan). Discordant results were assessed by re-testing samples with the screening test and secondary enzyme-linked immunosorbent assay. Remaining blood was stored at -70 °C. CD4 and VL testing was only conducted on stored blood samples from HIV-positive participants who consented to unspecified future blood analysis. During 2016–2017, stored samples were tested for HIV VL (COBAS AmpliPrep/COBAS TaqMan HIV-1 v2.0, Roche Molecular Diagnostics, USA) and CD4 cell counts (Vacutainer CD4 Stabilization Blood Collection System and FACSCount, BD Biosciences, USA). All laboratory testing and CD4 counting were carried out at the Provincial AIDS Center (PAC). Then the National Reference Laboratory at the National Institute of Hygiene and Epidemiology (NIHE) verified HIV testing performed at PAC by testing 5% of the HIV negative samples and 10% of the HIV positive samples using the testing protocol approved by Ministry of Health (MOH). All VL testing was tested at NIHE.

### Ethics

All participants provided written informed consent, including consent for storage of specimens and future unspecified laboratory analyses. Participants were compensated with 100,000 Vietnam dong (approximately US $ 5.00) for their time and travel expenses. A small monetary compensation of 50,000 Vietnam dong (approximately US$2.5) was also provided to participants for each additional participant they recruited. The study was reviewed and approved by the institutional review boards of the National Institute of Hygiene and Epidemiology, Vietnam; Family Health International, Vietnam; and the U.S. Centers for Disease Control and Prevention (CDC), USA^1^, and CDC investigators did not interact with human subjects or access identifiable data or specimens for research purposes. The study was conducted in accordance with the ethical principles of the Declaration of Helsinki.

### Data analysis

Behavioral and laboratory data were analyzed both by province and overall. The prevalence of HIV status awareness and ART use was calculated based on participants’ self-reported information. However, potential inaccuracies in self-reported HIV status due to social desirability bias may have influenced the results; for example, some participants might not disclose their HIV-positive status or unknown status even if they were on ART. To address this issue, participants with an undetectable viral load (< 20 copies/ml) were assumed to be aware of their HIV-positive status and using ART, regardless of their responses. Low CD4 cell count was defined as < 200 cells/mm^3^, however, we also evaluated CD4 cell count < 350 cells/mm^3^ – the threshold for ART initiation in 2013 as an alternative definition. RDS probability weights were calculated using RDS-Analyst software [7]. All weighted mean and weighted prevalence calculations, as well as our weighted logistic regression analyses, were conducted using the svy command set in STATA. Analyses accounted for clustering among participants enrolled by a common recruiter.

Logistic regression was used to explore factors associated with ART use among all study participants, as well as with low CD4 cell count among participants who were not using ART. All factors analyzed were considered for inclusion in our multivariate models and retained if one or more categories exhibited an adjusted p-value < 0.05. Province was considered an important variable for inclusion in all models and was retained regardless of its statistical significance due to potential regional heterogeneity in HIV epidemiology, health service availability, harm reduction coverage, and ART program implementation. These contextual differences could confound associations between individual-level factors and ART use or CD4 status.

## Results

A total of 287 male-PWID living with HIV (203 from Ho Chi Minh City and 84 from Can Tho) were included, and all had HIV VL and CD4 cell count results. The mean age was 33.8 years (see Table 1).

**Table 1:** Participant characteristics at the time of recruitment, among people who inject drugs with HIV in two southern provinces of Vietnam (*N* = 287)

Characteristic		Ho Chi Minh City (*N* = 203)	Can Tho (*N* = 84)	Overall (*N* = 287)
Age (years)	Weighted mean (95%CI)	33.9 (32.7–35.0)	31.5 (30.3–32.8)	33.8 (32.7–34.9)
Ethnicity	Kinh	92.8% (85.4–96.6)	96.0% (85.3–99.0)	92.9% (85.9–96.6)
	Non-Kinh	0.6% (0.1–3.9)	4.0% (1.0-14.7)	0.7% (0.1–3.2)
	Unknown	6.7% (3.0-14.1)	0.0%^	6.4% (2.9–13.6)
Living arrangement	Alone	8.4% (4.8–14.1)	2.0% (0.7–5.9)	8.1% (4.7–13.6)
	With wife/girlfriend	16.0% (10.7–23.3)	25.9% (15.4–40.2)	16.4% (11.2–23.4)
	With relatives	74.2% (65.9–81.1)	72.0% (58.1–82.7)	74.2% (66.2–80.8)
	Unknown	1.4% (0.5-4.0)	0.0%^	1.3% (0.5–3.8)
Education level	None/primary school	27.2% (20.5–35.2)	47.7% (32.4–63.4)	28.0% (21.5–35.6)
	Secondary school	56.6% (48.3–64.5)	35.9% (24.3–49.4)	55.7% (47.8–63.4)
	High school/university	16.2% (10.9–23.4)	16.4% (7.4–32.4)	16.2% (11.0-23.1)
Employment	Employed	59.0% (50.2–67.2)	73.1% (58.0-84.2)	59.5% (51.1–67.4)
	Unemployed	36.0% (28.2–44.6)	26.9% (15.8–42.0)	35.6% (28.1–43.9)
	Unknown	5.1% (2.2–11.4)	0.0%^	4.9% (2.1–10.9)
Individual monthly income (VND)	< 2.5 million	12.7% (8.1–19.2)	10.8% (5.9–18.7)	12.6% (8.2–18.8)
	2.5–4.5 million	38.3% (30.3–47.0)	56.6% (41.6–70.5)	39.0% (31.3–47.3)
	> 4.5 million	37.6% (29.6–46.3)	19.4% (10.1–34.2)	36.9% (29.2–45.2)
	Unknown	11.5% (6.2–20.2)	13.2% (6.6–24.7)	11.5% (6.4–19.8)
Self-reported HIV status prior to survey*	Positive	55.4% (47.1–63.4)	52.1% (35.7–68.1)	55.2% (47.3–62.9)
	Negative	6.8% (3.4–13.2)	14.4% (6.7–28.2)	7.1% (3.7–13.1)
	Do not know	37.8% (30.0-46.3)	33.5% (22.1–47.2)	37.6% (30.2–45.8)
Duration of injecting drug use (years)	< 5	20.3% (14.2–28.2)	33.5% (21.2–48.7)	20.8% (14.9–28.3)
	5–10	19.3% (13.8–26.4)	29.6% (18.3–44.1)	19.7% (14.3–26.5)
	> 10	56.1% (47.9–64.1)	36.9% (23.4–52.8)	55.4% (47.4–63.0)
	Unknown	4.3% (1.4–12.4)	0.0%^	4.1% (1.3–11.9)
Usual frequency of injecting drug use (per day)	Once	13.5% (8.3–21.3)	24.2% (14.1–38.1)	13.9% (8.9–21.3)
	Twice	59.7% (51.4–67.5)	59.0% (43.8–72.5)	59.7% (51.7–67.2)
	> Twice	26.7% (20.0-34.8)	16.9% (7.9–32.6)	26.4% (19.9–34.0)
Current use of methadone maintenance therapy	Using	6.8% (3.4–13.0)	3.8% (1.2–10.9)	6.7% (3.4–12.6)
	Not using	93.2% (87.0-96.6)	96.2% (89.1–98.8)	93.3% (87.4–96.6)
Ever attended drug rehabilitation^1^ – ever	Yes	64.3% (55.9–72.0)	58.9% (43.1–73.1)	64.1% (56.0-71.5)
	No	29.0% (22.1–37.0)	41.1% (26.9–56.9)	29.5% (22.8–37.2)
	Unknown	6.7% (3.0-14.1)	0.0%^	6.4% (2.9–13.6)
Attended drug rehabilitation in past 12 months	Yes	7.1% (3.9–12.7)	10.6% (4.9–21.4)	7.2% (4.1–12.5)
	No	84.6% (76.8–90.1)	87.7% (76.2–94.1)	84.7% (77.3–90.0)
	Unknown	8.3% (4.2–15.7)	1.7% (0.2–11.6)	8.0% (4.1–15.1)

In Vietnam, in the past drug users were mandatorily sent to rehabilitation centers for drug treatment as well as for job training. At present, if drug treatment in the community fails, then drug users are sent to rehabilitation centers for mandatory treatment

All values are presented as weighted percentages (95% confidence interval) unless otherwise indicated. *Participants with undetectable viral load were assumed to be aware of their HIV-positive status regardless of their survey responses; ^Confidence intervals were not calculated for exact 0% and 100% values; CI, confidence interval; VND, Vietnamese Dong

We found that just over half of participants (55.2%) were aware of their HIV-positive status. Importantly, 48.8% of participants, or 88.4% of those who were aware of their HIV positivity, were using ART. Furthermore, 81.6% of those on ART had undetectable VL. (Data not shown).

Older age (odds ratio [OR] 4.9 for participants > 40 years versus those < 30 years, 95% confidence interval [95% CI] 1.2–20.5) and current use of opioid substitution therapy (OST) (OR 12.7 versus non-use, 95% CI 1.1-141.7) were associated with a higher prevalence of ART use (see Table 2). Living alone (OR 0.1 versus living with relatives, 95% CI 0.0-0.5), living with one’s wife or girlfriend (OR 0.3 versus living with relatives, 95% CI 0.1–0.8), having a higher individual income (OR 0.2 for incomes > VND 4.5 million versus those < VND 2.5 million, 95% CI 0.1–0.7), and attending drug rehabilitation^2^ in the past 12 months (OR 0.2 versus not attending, 95% CI 0.1–0.8) were associated with a lower prevalence of ART use. The adjusted prevalence of ART use did not differ by province.

**Table 2:** Factors associated with antiretroviral therapy use among people who inject drugs with HIV in southern Vietnam (*N* = 287)

Characteristic	Raw overall *N*	Weighted % using ART	Univariate		Multivariate	
			OR (95%CI)	*p*	aOR (95%CI)	*p*
**Age (years)**						
< 30	92	33.7%	1.0		1.0	
30–40	156	54.3%	2.3 (1.1–5.1)	0.04	2.5 (1.1–5.8)	0.04
> 40	39	60.3%	3.0 (0.9–9.7)	0.07	4.9 (1.2–20.5)	0.03
**Living arrangement**						
Alone	21	21.2%	0.2 (0.1–0.9)	0.04	0.1 (0.0-0.5)	< 0.01
With wife/girlfriend	59	46.9%	0.8 (0.3–1.9)	0.58	0.3 (0.1–0.8)	0.02
With relatives	203	53.1%	1.0		1.0	
Unknown	4	0.0%	-		-	
**Education level**						
None/primary school	86	45.7%	1.0			
Secondary school	152	48.5%	1.1 (0.5–2.4)	0.77		
High school/university	49	54.8%	1.4 (0.5–3.9)	0.47		
**Employment**						
Employed	189	44.0%	1.0			
Unemployed	87	58.1%	1.8 (0.8–3.7)	0.14		
Unknown	11	39.0%	-			
**Individual monthly income (VND)**						
< 2.5 million	34	62.8%	1.0		1.0	
2.5–4.5 million	115	55.6%	0.7 (0.3–2.2)	0.59	0.6 (0.2-2.0)	0.37
> 4.5 million	107	31.9%	0.3 (0.1–0.8)	0.02	0.2 (0.1–0.7)	0.01
Unknown	31	64.3%	-		-	
**Duration of injecting drug use (years)**						
< 5	60	37.3%	1.0			
5–10	63	42.8%	1.3 (0.4–3.6)	0.67		
> 10	160	54.9%	2.0 (0.8-5.0)	0.12		
Unknown	4	52.6%	-			
**Usual frequency of injecting drug use (per day)**						
Once	41	57.1%	1.0			
Twice	168	46.1%	0.6 (0.2–1.8)	0.40		
> Twice	78	50.5%	0.8 (0.3–2.3)	0.64		
**Current use of methadone**						
Using methadone	19	91.1%	12.2 (1.7–89.0)	0.01	12.7 (1.1-141.7)	0.04
Not using methadone	268	45.7%	1.0		1.0	
**Ever attended drug rehabilitation** ^1^						
Yes	193	48.2%	1.0 (0.5-2.0)	0.94		
No	80	48.9%	1.0			
Unknown	14	53.8%	-			
**Attended drug rehabilitation in past 12 months**						
Yes	25	20.8%	0.3 (0.1-1.0)	0.06	0.2 (0.1–0.8)	0.02
No	244	49.8%	1.0		1.0	
Unknown	18	63.1%	-		-	
**Province**						
Ho Chi Minh City	203	48.9%	1.0		1.0	
Can Tho	84	46.6%	0.9 (0.4-2.0)	0.81	1.1 (0.4–2.7)	0.85

^1^ In Vietnam, in the past drug users were mandatorily sent to rehabilitation centers for drug treatment as well as for job training. At present, if drug treatment in the community fails, then drug users are sent to rehabilitation centers for mandatory treatment

Participants with missing data were included in all models but odds ratios for unknown categories are not presented. OR, odds ratio; aOR, adjusted odds ratio; CI, confidence interval; VND, Vietnamese dong

**Fig. 1:**
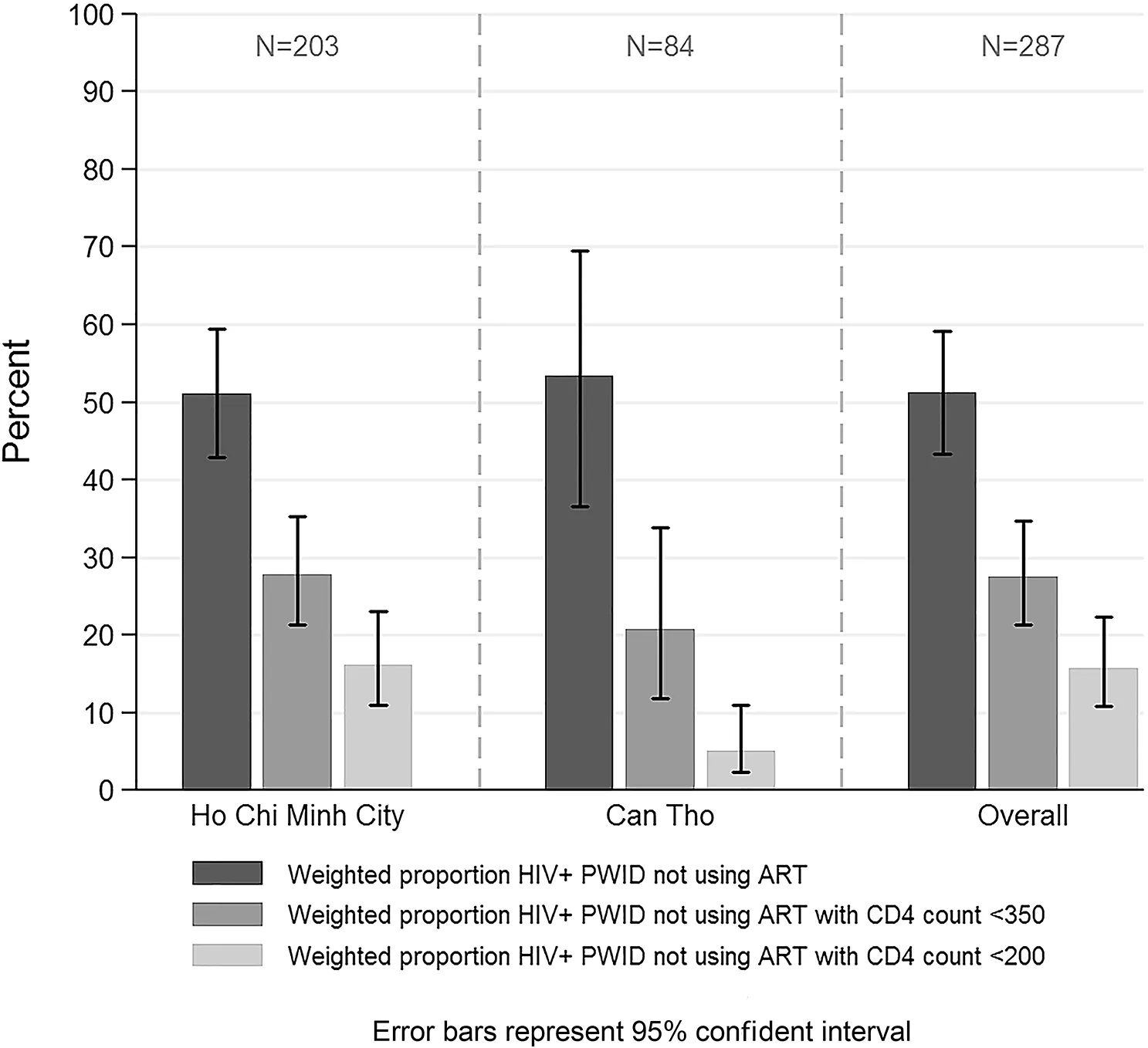
Weighted prevalence of antiretroviral therapy non-use and antiretroviral therapy non-use with low CD4 cell count among people who inject drugs with HIV in two southern provinces of Vietnam

Figure 1 also shows that 27.5% of PWID with HIV were not using ART with a CD4 cell count < 350 cells/mm^3^, and 15.7% were not using ART with a CD4 cell count < 200 cells/mm^3^. Among those not using ART, longer duration of injecting drug use (OR 6.3 for > 10 years versus < 5 years of injecting drug use, 95% CI 1.5–27.3) and province (OR 0.3 for Can Tho versus Ho Chi Minh City, 95% CI 0.1–0.7) were factors significantly associated with a CD4 cell count < 200 cells/mm^3^ (Table 3). Similar but attenuated results were seen with a CD4 cell count threshold of < 350 cells/mm^3^, with an OR of 2.4 for > 10 years of injecting drug use versus < 5 years (95% CI 0.8-7.0) and OR of 0.7 for Can Tho versus Ho Chi Minh City (95% CI 0.3–1.6).

**Table 3:** Factors associated with CD4 cell count < 200 cells/mm^3^ in individuals not using antiretroviral therapy among people who inject drugs living with HIV in two southern provinces of Vietnam (*N* = 153)

Characteristic	Raw overall *N*	Weighted % with CD4 < 200	Univariate		Multivariate	
			OR (95%CI)	*p*	aOR (95%CI)	*p*
**Age (years)**						
< 30	61	29.5%	1.0			
30–40	73	30.0%	1.0 (0.4–2.9)	0.97		
> 40	19	37.2%	1.4 (0.3–5.7)	0.63		
**Living arrangement**						
Alone	16	33.9%	1.3 (0.3–5.1)	0.70		
With wife/girlfriend	30	37.3%	1.5 (0.4–6.4)	0.58		
With relatives	103	28.2%	1.0			
Unknown	4	34.9%	-			
**Education level**						
None/primary school	50	38.6%	1.0			
Secondary school	81	29.4%	0.7 (0.2-2.0)	0.46		
High school/university	22	18.9%	0.4 (0.1–1.7)	0.21		
**Employment**						
Employed	112	26.9%	1.0			
Unemployed	38	34.1%	1.4 (0.5-4.0)	0.52		
Unknown	3	54.4%	-			
**Individual monthly income (VND)**						
< 2.5 million	14	19.1%	1.0			
2.5–4.5 million	59	44.7%	3.4 (0.4–29.9)	0.27		
> 4.5 million	67	21.6%	1.2 (0.2–8.8)	0.88		
Unknown	13	39.8%	-			
**Duration of injecting drug use (years)**						
< 5	39	8.3%	1.0		1.0	
5–10	39	30.5%	4.8 (1.0-22.7)	0.05	5.0 (1.0-23.6)	0.04
> 10	73	37.0%	6.5 (1.5–27.6)	0.01	6.3 (1.5–27.3)	0.01
Unknown	2	100.0%	-		-	
**Frequency of injecting drug use (per day)**						
Once	24	51.2%	1.0			
Twice	92	26.8%	0.3 (0.1–1.3)	0.11		
> Twice	37	30.7%	0.4 (0.1-2.0)	0.28		
**Ever attended drug rehabilitation**						
Yes	101	31.2%	1.4 (0.5–3.9)	0.54		
No	49	24.7%	1.0			
Unknown	3	54.4%	-			
**Attended drug rehabilitation in past 12 months**						
Yes	17	25.2%	0.8 (0.2–3.9)	0.78		
No	133	29.7%	1.0			
Unknown	3	54.4%	-			
**Province**						
Ho Chi Minh City	103	31.5%	1.0		1.0	
Can Tho	50	9.5%	0.2 (0.1–0.6)	< 0.01	0.3 (0.1–0.7)	0.01

Participants with missing data were included in all models but odds ratios for unknown categories are not presented. OR, odds ratio; aOR, adjusted odds ratio; CI, confidence interval; VND, Vietnamese dong

## Discussion

This study provides important insights into ART use and immunologic status among PWID living with HIV in two urban centers of Vietnam. Just over half of participants were aware of their HIV-positive status, and while most of those engaged in care achieved viral suppression, a substantial proportion remained undiagnosed or untreated. These findings underscore that HIV case finding remains a critical gap in reaching the UNAIDS 95–95–95 targets.

Our finding that more than half of participants were aware of their HIV-positive status represents a modest increase compared with earlier IBBS estimates [8]. This increase was due to the re-classification of those with an undetectable viral load who reported a negative or unknown status. Nevertheless, the fact that approximately half of PWID living with HIV in Ho Chi Minh City and Can Tho did not know they are HIV-positive remains a concern. This is consistent with the 2014 UNAIDS report which estimated that only 48% of people living with HIV know their status worldwide [9]. Major of those who were aware of their HIV positivity, were liked to ART. Importantly, most of those on ART had undetectable VL. These findings indicate that PWID with HIV in Vietnam are likely linked to ART if they are aware of HIV status and do well once they are engaged in treatment.

An association between older age and higher prevalence of ART use has been demonstrated elsewhere [10] and may be indicative of those with a longer duration of HIV infection being more likely to have sought care. We also found that being engaged in OST was highly associated with ART use. This highlights the effectiveness of the linkage between OST clinics and HIV testing sites in Vietnam, and reinforces the findings of a recent systematic review which found that OST was associated with a 54% increase in ART coverage among PWID [11]. Conversely, those with a recent history of attending a drug rehabilitation center had a low probability of ART use. Although admission to drug rehabilitation is typically associated with mental, social and financial instabilities [12] which may complicate ART use, it can be important to explore all potential opportunities for improving HIV care engagement. While significant barriers exist in these facilities, efforts to expand evidence-based approaches such as OST and integrated HIV services remain critical in ensuring effective and equitable access to HIV testing and treatment for PWID. Our data suggest that more could be done to utilize this opportunity in Ho Chi Minh City and Can Tho. PWID living alone or with their partner were less likely to be on ART than those living with relatives. This is consistent with other studies which have shown people living with HIV are more likely to use and adhere to ART when they have strong social support at home [13, 14]. Importantly, this association appears to be contingent upon the disclosure of one’s HIV status within the household or among friend and acquaintance networks [14]. Those with a high individual income also had a lower rate of ART use. This could be related to the ongoing stigma suffered by people living with HIV and PWID in Vietnam [15] and concern that using ART use will lead to one’s HIV status becoming more widely known. Well-paid PWID may feel that the benefit of ART is not worth the potential loss of income associated with being identified as HIV-positive.

In a large Asian HIV cohort, late ART initiation (defined as a CD4 cell count < 200 cells/mm^3^ or an AIDS diagnosis prior to starting ART) declined from 82.4% in 2007 to 36.3% after 2011 [4]. While we report a lower proportion of PWID with HIV not using ART with a CD4 count < 200 cells/mm^3^, our numerator did not include participants who had already started ART with a low CD4 count nor those with a history of AIDS. Unfortunately, we did not have data available on prior AIDS diagnoses or CD4 cell count at ART initiation.

Long-term PWID are more likely than short-term ones to have a history of illness due to advanced symptoms of HIV infection and are less likely to seek help for their drug addiction [16]. These factors could impair the ability of long-term PWID to recognize and act upon advanced symptoms of HIV infection, resulting in them seeking care only when they are severely unwell. Unfortunately, as it has now been well documented, late ART initiation leads to a substantially elevated risk of morbidity and mortality [17]. Compared with other provinces, the PWID population in Ho Chi Minh City comprises a large number of long-term users [2]. However, even after adjusting for the duration of injecting drug use, we found PWID living with HIV in Can Tho had lower adjusted odds of having a low CD4 count compared to those in Ho Chi Minh City. It is uncertain what is driving this association, but it highlights an important issue for health care providers and policy makers in Ho Chi Minh City.

The marked decline in the proportion of new HIV infections attributable to injecting drug use in Vietnam over the past decade likely reflects the substantial scale-up of evidence-based harm-reduction and treatment interventions. Expanded coverage of needle and syringe programs, OST, and antiretroviral treatment among PWID has been shown to reduce HIV incidence in this population. In parallel, improved HIV testing and earlier treatment initiation may have reduced onward transmission among PWID. At the same time, the relative contribution of injecting drug use to new HIV infections has declined as sexual transmission, particularly among men who have sex with men, has become a more prominent driver of the epidemic. Together, these shifts underscore both the success of harm-reduction programs for PWID and the need to sustain and adapt HIV prevention strategies across populations at higher risk for HIV [3, 5, 18]. 

The cross-sectional design precludes causal inference between identified factors and ART use or CD4 status. Associations observed should therefore be interpreted as correlational rather than causal. Our study population only comprised of PWID living in Ho Chi Minh City and Can Tho hence the results may not be generalizable to other parts of Vietnam. Much of our data on risk behaviors were collected via self-reporting, which is subject to recall and influenced by social norms. Our efforts to mitigate such influences included obtaining informed consent, intensive training for staff related to interview techniques to establish trust with the participants, and triangulating responses with multiple questions to identify discrepancies. We attempted to reduce misclassification by reclassifying participants with undetectable viral load as being aware of their HIV-positive status and on ART. We analyzed factors associated with ART use and low CD4 cell count by combining provincial data based on the assumption that associations were similar across provinces. Pooling data increased the study’s statistical power, allowing for more robust inferences that could be more readily translated into policy and practice. Although these data were collected in 2013, there has since been significant expansion of HIV testing, ART, and medication-assisted treatment services for PWID in Vietnam. Nevertheless, the risk factors identified in this analysis remain important considerations, as they continue to inform current and future interventions aimed at reducing late ART initiation and improving treatment outcomes among PWID. Despite these limitations, the study offers valuable insights into gaps in HIV diagnosis, treatment uptake, and immunologic status among PWID, and identifies subgroups that may benefit from targeted interventions to prevent late ART initiation and advanced immunosuppression.

## Conclusion

Just over half of PWID were aware of their HIV-positive status, and among those, nearly nine out of ten were receiving antiretroviral therapy (ART), with most achieving undetectable viral load. ART use was associated with older age and engagement in OST, whereas certain social and structural factors including living arrangements, income, and recent attendance at drug rehabilitation facilities were associated with lower treatment uptake. Among those not on ART, a notable proportion had severely low CD4 cell counts, with longer durations of injecting drug use strongly associated to advanced immunosuppression. Although these data were collected in 2013, the findings provide important historical context and underscore persistent barriers to timely HIV diagnosis and treatment among PWID. To achieve the UNAIDS 95:95:95 goals, expanding HIV testing including case finding effort in community and closed settings, and treatment services tailored to meet the need of PWID may increase the awareness of HIV status and engagement in HIV care among this population. PWID with HIV who reside in mandatory rehabilitation centers are an accessible group who could be better linked to HIV care. These insights remain relevant for informing HIV program design aimed at promoting early ART initiation improving outcomes among PWID with HIV in Vietnam and similar settings.

## Supplementary Information

Below is the link to the electronic supplementary material.

Supplementary Material 1

## Data Availability

The dataset analyzed in this study is owned by the Vietnam Ministry of Health and the National Institute of Hygiene and Epidemiology. Data are available upon reasonable request and with permission from the Ministry of Health.

## Footnotes

1 See 45 Code of US Federal Regulations (C.F.R.), part 46; 21 C.F.R. part 56.

2 In Vietnam, in the past drug users were mandatorily sent to rehabilitation centers for drug treatment as well as for job training. At present, if drug treatment in the community fails, then drug users are sent to rehabilitation centers for mandatory treatment.

## Abbreviations

AIDS: Acquired immunodeficiency syndrome
ART: Antiretroviral therapy
BBS: Behavioral and biologic survey
CDC: U.S. centers for disease control and prevention
CI: Confidence interval
HIV: Human immunodeficiency virus
IBBS: Integrated behavioral and biologic survey
IDU: Injecting drug user
MMT: Methadone maintenance therapy
MOH: Ministry of health
MSM: Men who have sex with men
OR: Odds ratio
PAC: Provincial AIDS center
PEPFAR: U.S. president’s emergency plan for AIDS relief
PLHIV: People living with HIV
PWID: People who inject drugs
RDS: Respondent-driven sampling
STI: Sexually transmitted infection
UNAIDS: Joint united nations programme on HIV/AIDS
VL: Viral load
VND: Vietnamese dong
WHO: World Health Organization
