# Supplementary material for: Antiretroviral therapy use and CD4 cell count among people who inject drugs living with HIV in southern Vietnam
Source: BMC Infect Dis. 2026 Jul 2;26:1703. doi: 10.1186/s12879-026-13180-2 (PMC13599271; doi:10.1186/s12879-026-13180-2)
Supplement: Supplementary file 1 [file 12879_2026_13180_MOESM1_ESM.doc]

###### QUESTIONNAIRE FOR MALE INJECTION DRUG USERS (IDU)

***IBBS round III, 2012***

001 QUESTIONNAIRE IDENTIFICATION NUMBER	|___|___|___|___|___|___|___|

002 CITY CODE	|___|___|

003 RECRUITMENT INFORMATION

COUPON NUMBER (*Only use for respondent-driven sampling)*

*|___|___|___|___|___|___|___|*

CLUSTER NUMBER (*Only use for Time Location Sampling)*

*Cluster number	|___|___|*

*Sequence number is invited subjects in clusters*	|___|___|

004. NAME OF INTERVIEWER: _____________________Signature ____________________

005. DATE OF INTERVIEW: __/ ____ / ____

006. CHECKED BY LOCAL SUPERVISOR: Name: ____________Signature _____________

DATE CHECKED: __/ ____ / ____

007. CHECKED BY NATIONAL SUPERVISOR: Name: ____________Signature __________

DATE CHECKED: __/ ____ / ____

**DATA MANAGEMENT**

| For project staff only | Check and cleaning by | Coding by | 1st data entry by | 2nd data entry by | Check by |
| --- | --- | --- | --- | --- | --- |
| **Name** |  |  |  |  |  |
| **Date** |  |  |  |  |  |

**SECTION 1: BACKGROUND CHARACTERISTICS**

| **No.** | **Questions** | **Coding of answers** | | **Skip to** |
| --- | --- | --- | --- | --- |
| Q101 | In what month and year were you born? | Month \|__\|__\|  Don’t remember 99  Year \|__\|__\|__\|__\|  Don’t remember 9999 | |  |
| Q102 | What is the highest level of education you have finished?  *(Circle only* ***one*** *choice)* | Illiterate 1  Primary (Grade 1-5) 2  Secondary school (Grade 6-9) 3  High school (Grade 10 – 12) 4  College, university (>12) 5 | |  |
| Q103 | How long have you lived here in (name of this city/province)? | \|__\|__\| years  \|__\|__\| months  *Record 01 if less than 1 month*  Don’t remember 99 | |  |
| Q104 | What is your ethnicity? | Kinh 1  Hoa 2  Khmer 3  Tay 4  H’mong 5  Other 6  (Specify) …………………………………..… | |  |
| Q105 | ***During the last 12 months,*** have you been away from **this** city/province for one month or more continuously? | Yes 1  No 2  Don’t remember 9 | |  |
| Q106 | ***Currently***, who are you living with?  *(Circle only* ***one*** *choice)* | Alone 1  With wife/ Girlfriend 2  With relatives 3  With friends 4  Unsettled 5  Other (specify) 6  ………………………………………….. | |  |
| Q107 | Have you ever been married? | Never 1  Currently married 2  Divorced 3  Separated 4  Widowed 5 | |  |
| Q 108 | How many children do you have? | [__\|__] children | |  |
| Q109 | ***During the last month,*** how often have you had beer/alcoholic drinks? | \|__\|__\| Number of days | |  |
| Q110 | Are you currently employed? | Yes 1  No 2  No response 9 | | → Q112  → Q112 |
| Q111 | ***Currently***, what occupations do you have to support yourself?  *Probe: anything else?*  ***Don’t read choices. Only probe and circle all that apply*** | Farmer  Government employee Entertainment employee Sales/office clerk  Business person Student  Self-employed (xe om, vendor, etc.)  Illegal activities (gambling, deal dealing, etc.)  Others (specify)  ………………………… | **Y N**   1 2 1 2 1 2 1 2 1 2 1 2 1 2 1 2   1 2 |  |
| Q112 | ***During the last 12 months***, what was your FAMILY monthly income?  *(Enter total income from all family members)* | \|__\|__\|__\|,\|__\|__\|__\|,\|__\|__\|__\| VND  Don’t remember/No response 99 | |  |
| Q113 | ***During the last 12 months***, what was your monthly income?  *(Enter total income from all sources)* | \|__\|__\|__\|,\|__\|__\|__\|,\|__\|__\|__\| VND  Don’t remember/No response 99 | |  |
| Q114 | Have you ever been in a drug rehabilitation center (06 center)? | Yes 1  No 2  No response 9 | | → Q201  → Q201 |
| Q115 | How many times you have been in a drug rehabilitation center (06 center)? | \|__\|__\|__\| time  Don’t remember/No response 99  Ever 00 | | → Q201  → Q201 |
| Q116 | ***The last time*** you were in 06 center, how did you get admitted to the center?  *(Do not read responses. Circle only one choice)* | Forced by local authority 1  Sent by the family 2  Self-referred 3  Other (specify) ……………………………4 | |  |
| Q117 | When were you ***last released*** from 06 center? | Month [__\|__]  Don’t remember 99  Year [__\|__\|__\|__]  Don’t remember 9999 | |  |

###### SECTION 2: DRUG USE

I WILL NOW ASK YOU ABOUT YOUR DRUG USING BEHAVIOR.

| **No.** | **Questions** | **Coding of answers** | **Skip to** |
| --- | --- | --- | --- |
| Q201 | In what month and year did you start using drugs? | Month \|__\|__\|  Don’t remember 99  Year \|__\|__\|__\|__\|  Don’t remember 9999 |  |
| Q202 | In what month and year did you start injecting drugs? | Month \|__\|__\|  Don’t remember 99  Year \|__\|__\|__\|__\|  Don’t remember 9999 |  |
| Q203 | What type of drug did you start injecting first? | Opium “Black” 01  Heroin “White” 02  Sedatives (Valium, Seduxen, Benzoate) 03  Ketamine 04  Cocaine 05  Other 06  (Specify)…………………………………… |  |
| Q204 | What types of drugs have you used?  ***Circle all that apply.***  ***If the respondent has not used drugs, then do not follow up with the question for “Past 1 month.*** | **Ever**  **Y N**  Opium 1 2  Heroin 1 2  Marijuana 1 2  Ecstasy (etc.) 1 2  Ice/Methamphetamine (etc.) 1 2  Inhalants 1 2  Ketamine 1 2  Magic mushrooms 1 2  Cocaine 1 2  Sedatives (Valium, Seduxen, Benzoate) 1 2  Other 1 2  (Specify)……….……………………………… | **Last month**  **Y N**  1 2  1 2  1 2  1 2  1 2  1 2  1 2  1 2  1 2  1 2  1 2 |
| Q205 | In the past 48 hours, have you used opiate substances? These include opium and heroin. | Yes 1  No 2 |  |
| Q206 | In the past 48 hours, have you used amphetamine type substances? These include methamphetamine (ice), ecstasy, or cocaine. | Yes 1  No 2 |  |
| Q207 | What types of drugs have you injected?  ***Circle all that apply.***  ***If the respondent has not used drugs, then do not follow up with the question for “Last month”*** | **Ever**  **Y N**  Opium “Black” 1 2  Heroin “White” 1 2  Sedatives (Valium, Seduxen, Benzoate) 1 2  Ecstasy (etc.) 1 2  Ice/Methamphetamine (etc.) 1 2  Ketamine 1 2  Cocaine 1 2  Other 1 2  (Specify)…………………………………… ….. | **Last month**  **Y N**  1 2  1 2  1 2  1 2  1 2  1 2  1 2  1 2 |
| Q208 | ***During the last month***, how many times in a day have you injected drugs? | \|__\|__\| times  Don’t remember 99  If less than 1 time per day, code as 00 |  |
| Q209 | ***On average***, how much do you spend per day on heroine? | \|__\|__\|__\|, \|__\|__\| __\|, \|__\|__\|__\| VND |  |
| Q210 | ***In the last week,*** how often did to you go to injecting sites or places where many people gather to inject? | \|__\|__\| times  Don’t remember 99  If Don’t go , code as 00 | **→ Q212** |
| Q211 | ***If any,*** how many sites did you go to in the past week? | \|__\|__\| sites  Don’t remember 99 |  |
| Q212 | ***If any,*** what kind of place?  ***Don’t read choices. Only probe and circle all that apply*** | Y N  Streets, alleys, parks, cemetery, railroad tracks 1 2  Railroad tracks 1 2  Someone’s home 1 2  Bar/restaurants 1 2  Other 1 2  (Specify)…………………………………… |  |
| Q213 | **If yes,** how many people are there in that place? | Streets, alleys, parks, cemetery \|__\|__\|__\|  Railroad tracks \|__\|__\|__\|  Someone’s home \|__\|__\|__\|  Bar/restaurants \|__\|__\|__\|  Other \|__\|__\|__\|  (Specify)…………………………………… |  |
| Q214 | **During the last week**, where were you when you injected?  *(Do not read choices aloud. Circle only one answer)* | At home 1  At boyfriend’s/girlfriend’s home 2  At injecting partner’s home 3  At a public place (street/park) 4  At a shooting venue for IDUs 5  At drug dealer venue 6  With FSW 7  Other (specify) …………………….8 |  |
| Q215 | ***In the last ten times you acquired clean needles & syringes***, how many times did you purchase them and how many times did you obtain them for free from PE and/or NSP or other sources? | Number of times purchased \|__\|__\|  Number of times obtained \|__\|__\|  Don’t know/no response 99 |  |
| Q216 | How many times have you tried to quit using heroine? | \|__\|__\| Number of times  Don’t know/no response 99  If No , code as 00 | **→ Q301**  **→ Q301** |
| Q217 | What forms of drug treatment have you tried?  ***Circle all that apply*** | Y N  Detoxification (sedatives therapies, lotus, acupuncture, cedemex, Hufusa, other traditional medicines) 1 2  Methadone Medication assisted therapy 1 2  Suboxone Medication assisted therapy 1 2  Relapse prevention medication (naltrexone)  1 2  Naltrexone therapy 1 2  Psychosocial support (counseling groups, network) Community support 1 2  Residential support 1 2  “Cai bo” 1 2  Don’t know/don’t remember/no response 99 |  |

##### SECTION 3: NEEDLE SHARING BEHAVIORS

I WILL NOW ASK YOU ABOUT YOUR NEEDLE SHARING BEHAVIOR.

| **No.** | **Questions** | **Coding of answers** | **Skip to** |
| --- | --- | --- | --- |
| Q301 | When you injected, have you ever shared needles/syringes with others?  *(Explain: sharing needles mean using needles/syringes that had previously been used by someone else OR giving needles you had just finished using to someone else)* | Yes 1  No 2  No response 9 | **→Q307**  **→Q307** |
| Q302 | ***During the last 6 months,*** when you injected, how often have you used needles/syringes **that had previously been used** by someone else, OR **given syringes and needles that you *had already used*** to someone else? | Always 1  Occasionally 2  Never 3  No response 9 | **→Q307**  **→Q307** |
| Q303 | ***During the last month,*** how often have you given needles/syringes that ***you had already used*** to someone else? | Always 1  Occasionally 2  Never 3  No response 9 |  |
| Q304 | ***During the last month,*** how often have you used needles/syringes that had ***previously been used*** by someone else? | Always 1  Occasionally 2  Never 3  No response 9 |  |
| Q305 | ***During the last month,*** how often did you clean the needle/syringe that had ***previously been used*** by someone else before you used it again? | Always 1  Occasionally 2  Never 3  No response 9 | **→Q307**  **→Q307** |
| Q306 | ***If yes***, how did you usually clean the needle/syringe?  *(Circle all that apply)* | Y N  Cold water 1 2  Hot water 1 2  Bleach 1 2  Alcohol 1 2  Others (specify) 1 2  ……………………………… |  |
| Q306a | ***If yes,*** have you ever used the insulin syringe?  ***(Show one for example)*** | Yes 1  No 2  Don’t remember 3 |  |
| Q306b | What type of syringe do you prefer? | Normal syringe 1  Insulin syringe 2  Prefer normal syringe as much as insulin syringe 3  No idea 4 |  |
| Q306c | ***During the last month,*** what type of syringe do you usually use? | Normal syringe 1  Insulin syringe 2 |  |
| Q307 | ***In the last 6 months,*** how often did you share drug solution?  *(Sharing solution means mixing the solution and dividing among the users)* | Always 1  Occasionally 2  Never 3  No response 9 |  |
| Q308 | ***In the last 6 months,*** how often did you share water and containers to mix injection solution? | Always 1  Occasionally 2  Never 3  No response 9 |  |
| Q309 | ***In the last 6 months,*** how often have you had difficulty accessing new needles? | Always 1  Occasionally 2  Never 3  No response 9 |  |
| Q310 | ***During the last 12 months,*** have you ever injected drugs in another city/province? | Yes 1  No 2  Don’t remember 8  No response 9 | **→ Q401**  **→ Q401**  **→ Q401** |
| Q311 | ***If yes,*** in which other city/provinces did you inject, including city/provinces in other countries?  *(Specify name of province, city, or country)* | ................................................................................................................................................................................................................................................ |  |

**SECTION 4: BEHAVIORS AT THE LAST INJECTION**

I WILL NOW ASK YOU ABOUT YOUR LAST INJECTION

| **No.** | **Questions** | **Coding of answers** | **Skip to** |
| --- | --- | --- | --- |
| Q401 | ***The last time you injected***, where were you when you injected?  *(Do not read choices aloud. Circle only one answer)* | At home 1  At boyfriend’s/girlfriend’s home 2  At injecting partner’s home 3  At a public place (street/park) 4  At a shooting venue for IDUs 5  At drug dealer venue 6  With FSW 7  Other (specify) …………………….8 |  |
| Q402 | ***The last time you injected***, how many people did you get high with? | \|__\|__\| injectors  Don’t remember/No response 99 |  |
| Q403 | ***if Q301=1, ask:***  ***The last time you injected***, did you share needle, solution, water or container to mix the solution?  *(Explain: sharing means using the above items that had previously been used by someone else OR giving the above items you had just finished using to someone else)* | Yes 1  No 2  Don’t know 9 | **→ Q501**  **→ Q501** |
| Q404 | ***The last time you injected***, how many other injectors shared the same needle/syringe, solution, water or container to mix the solution? | \|__\|__\| injectors  Don’t remember/No response 99 |  |
| Q405 | Of those *(READ RESPONSE FOR Q404),* how many do you know are HIV positive? | \|__\|__\| persons  Don’t remember/No response 99 |  |

**SECTION 5: IDU NETWORK**

I WILL NOW ASK YOU ABOUT THE NUMBER OF PEOPLE YOU KNOW SIMILAR TO YOURSELF.

| **No.** | **Questions** | **Coding of answers** | **Skip to** |
| --- | --- | --- | --- |
| Q501 | How many injecting drug users do you know who also know you (i.e., you know their name and they know your name)?  ***(Don’t count the people who are in the drug rehabilitation center or dead)*** | \|__\|__\|__\| persons |  |
| Q502 | How many of those *(READ RESPONSE TO Q501)* are male 18 years old and over? | \|__\|__\|__\| persons |  |
| Q503 | ***During the last 2 weeks***, of those *(READ RESPONSE TO Q502)* how many have you seen? | \|__\|__\|__\| persons |  |
| Q504 | Of all the injecting drug users you know how many:   a) Inject mainly at home? b) Are homeless? c) Are female? | \|__\|__\|__\| persons  \|__\|__\|__\| persons  \|__\|__\|__\| persons |  |
| Q505 | According to you, who knows you are drug injector? | Your wife/girlfriend 1 2  Your parents 1 2  Your child 1 2  Your sisters/brothers 1 2  Relative 1 2  Friend 1 2  Colleagues 1 2  Neighbors 1 2  Community authority 1 2 |  |

##### SECTION 6: SEXUAL HISTORY: NUMBERS AND TYPES OF SEXUAL PARTNERS

I WILL NOW ASK YOU QUESTIONS RELATED TO YOUR SEXUAL ACTIVITIES.

| **No.** | **Questions** | **Coding of answers** | **Skip to** |
| --- | --- | --- | --- |
| Q601 | Have you ever had vaginal or anal sex? | Yes 1  No 2 | **→Q1101** |
| Q602 | How old were you when you had sexual intercourse for ***the first time***? | Age \|__\|__\|  Don’t remember 99 |  |
| Q603 | ***During the last 12 months***, how many different partners did you have sexual intercourse with?  *(****Read aloud:*** *Please think about this question for a while in order to give us the most correct answer. Your answer will be kept confidential.)* | Number of sex partners in past year \|__\|__\|  *(If none, enter* 00)  Don’t remember 99 | **→Q1101**  **→Q1101** |
| Q604 | ***During the last 12 months***, among your sexual partners, how many were:  ***Interviewer must read the following:***  **Wife/girlfriend** are those you have regular sex and do not pay money (or you are married/live with)  **Female sex workers**: who you have sex with and pay money  **Non-regular partners**: Any other sexual partners not including wife/girl friends or female sex workers  **Male partners** with whom you had anal sex | 604.1 **Wife/girlfriend** \|__\|__\|  Don’t remember 99  604.2 **Female sex workers** \|__\|__\|  Don’t remember 99  604.3 **Non-regular partners** \|__\|__\|  Don’t remember 99  604.4 **Male partners** \|__\|__\|  Don’t remember 99  *(Note: Check number of sexual partners in Q603 to ensure the numbers match)* |  |
| Q605 | ***In the last 12 months,*** with whom do you have sex most often? | Wife/girlfriend 1  Female sex workers 2  Non-regular partners 3  Male partners 4 |  |
| Q606 | ***The last time you had sex,*** with whom did you have sex? | Wife/girlfriend 1  Female sex worker 2  Casual partner 3  Male sex partner 4  No response / don’t remember 9 |  |

##### SECTION 7: SEXUAL HISTORY: WIFE/GIRL FRIEND

This section is used if the answer 604.1 is 1 or more than 1 or Don’t remember

| **No.** | **Questions** | **Coding of answers** | **Skip to** |
| --- | --- | --- | --- |
| Q701 | ***During the last month,*** how many times have you had vaginal or anal sex with your wife/ girlfriend? | Number of times \|__\|__\|  Don’t remember 99 |  |
| Q702 | ***The last time you had sex*** with your wife/girlfriend, did you use a condom?  *(****Read aloud:*** *Please think about this question for a while in order to give us the most correct answer. Your answer will be kept confidential.)* | Yes 1  No 2  Don’t remember 9 | **🡪 Q704**  **🡪 Q704** |
| Q703 | ***If yes***, who suggested that a condom be used that time? | Myself 1  My partner 2  Joint decision 3  Don’t remember 9 |  |
| Q704 | ***During the last 12 months,*** how often have you used condoms with your wife/girlfriend?  *If the response is “Always” or “Never”, then check response to Q702.* | Always 1  Occasionally 2  Never 3  Don’t remember 9 | **🡪 Q706**  **🡪 Q706** |
| C705 | What are your reasons for using a condom during sex with your wife/girlfriend? | **Yes No**  Contraception 1 2  Prevent disease transmission 1 2  Cleanliness 1 2  Increase staying power 1 2  Other 1 2  (Specify …………………………) |  |
| Q706 | ***During the last 12 months***, do you think your wife and /or girl friend has injected drugs? | Yes 1  No 2  Don’t know 9 |  |

##### SECTION 8: SEXUAL HISTORY: FEMALE SEX WORKERS

This section is used if the answer 604.2 is 1 or more than 1 or Don’t remember

| **No.** | **Questions** | **Coding of answers** | **Skip to** |
| --- | --- | --- | --- |
| Q801 | ***During the last month,*** how many times have you had vaginal or anal sex with female sex workers? | Number of times \|__\|__\|  Don’t remember 99 |  |
| Q802 | ***The last time you had sex*** with a female sex worker, was a condom used?  *(****Read aloud:*** *Please think about this question for a while in order to give us the most correct answer. Your answer will be kept confidential.)* | Yes 1  No 2  Don’t remember 9 | **🡪 Q804**  **🡪 Q804** |
| Q803 | ***If yes***, who suggested that a condom be used that time? | Myself 1  My partner 2  Joint decision 3 |  |
| Q804 | ***During the last 12 months,*** how often have you used condoms with female sex workers?  *If the response is “Always” or “Never”, then check response to Q802.* | Always 1  Occasionally 2  Never 3  Don’t remember 9 | **🡪Q 806**  **🡪Q 806** |
| Q805 | What are your reasons for using a condom during sex with female sex workers? | **Yes No**  Contraception 1 2  Prevent disease transmission 1 2  Cleanliness 1 2  Increase staying power 1 2  Other 1 2  (Specify …………………………) |  |
| Q806 | ***During the last 12 months***, do you think any of female sex workers that you have sex with has injected drugs? | Yes 1  No 2  Don’t know 9 |  |

##### SECTION 9: SEXUAL HISTORY: NON-REGULAR PARTNERS

This section is used if the answer 604.3 is 1 or more than 1 or Don’t remember

| **No.** | **Questions** | **Coding of answers** | **Skip to** |
| --- | --- | --- | --- |
| Q901 | ***During the last month,*** how many times have you had vaginal or anal sex with your non-regular sexual partners (not including wife/girlfriend or female sex workers) | Number of times \|__\|__\|  Don’t remember 99 |  |
| Q902 | ***The last time you had sex*** with a non-regular partner, was a condom used?  *(****Read aloud:*** *Please think about this question for a while in order to give us the most correct answer. Your answer will be kept confidential.)* | Yes 1  No 2  Don’t remember 9 | **🡪Q 905**  **🡪 Q905** |
| Q903 | ***If yes***, who suggested that a condom be used that time? | Myself 1  My partner 2  Joint decision 3 |  |
| Q904 | ***During the last 12 months***, how often have you used condoms with your non-regular sexual partners?  *If the response is “Always” or “Never”, then check response to Q902.* | Always 1  Occasionally 2  Never 3  Don’t remember 9 | **🡪 Q906**  **🡪 Q906** |
| Q905 | What are your reasons for using a condom during sex with your non-regular sexual partners? | **Yes No**  Contraception 1 2  Prevent disease transmission 1 2  Cleanliness 1 2  Increase staying power 1 2  Other 1 2  (Specify …………………………) |  |
| Q906 | ***During the last 12 months***, do you think any non-regular sex partner has injected drugs? | Yes 1  No 2  Don’t know 9 |  |

# SECTION 10: CONDOM USE

# Check section 7, 8, 9. Ask the people who reported had used condom

| **No.** | **Questions** | **Coding of answers** | **Skip to** |
| --- | --- | --- | --- |
| Q1001 | ***In the last 6 months,*** can you always get a condom when you need one? | Always 1  Occasionally 2  Never 3  No response 9 |  |
| Q1002 | ***In the last ten times you acquired condoms***, how many times did you purchase them and how times did you obtain them for free? | Number of times purchased \|__\|__\|  Number of times obtained \|__\|__\|  Don’t remember 99 |  |
| Q1003 | Where do you usually get condoms?  ***(Do not read choices aloud. Only probe for more responses. Circle all that apply)*** | Yes No  Pharmacy 1 2  Grocery store 1 2  Healthcare establishment 1 2  Bar/restaurant/hotel 1 2  Peer educator 1 2  Outreach health workers 1 2  Drop-in center 1 2  Harm reduction boxes 1 2  Others (specify) 1 2  ……………………………… |  |
| Q1004 | What type of condom do you usually use?  ***(Show the picture of condoms and*** ***Circle all that apply)*** |  |  |
| Q1005 | Which one you prefer? |  |  |

##### SECTION 11: CO-MORBIDITY

# I WILL NOW ASK YOU ABOUT SEXUALLY TRANSMITTED INFECTIONS & OTHER ILLNESSES.

| **No.** | **Questions** | **Coding of answers** | | | | **Skip to** |
| --- | --- | --- | --- | --- | --- | --- |
| Q1101 | ***During the last 12 months,*** have you had unusual genital discharge? | Yes 1  No 2  Don’t know 9 | | | |  |
| Q1102 | ***During the last 12 months,*** have you had genital sores or ulcers? | Yes 1  No 2  Don’t know 9 | | | |  |
| ***If both answers in Q1201 and Q1202 are “No”, SKIP to Q1104*** | | | | | | |
| Q1103 | ***a. The last time*** you had genital sore, ulcer or unusual discharge what did you do?  ***(Read list aloud. Circle all that apply)***   a. Went to government health facilities for examination and treatment b. Went to private health facilities for examination and treatment c. Went to pharmacy to buy drugs d. Went to traditional healer for examination and treatment e. Cured myself at home f. Told my regular sexual partners about the symptoms g. Stopped having sexual intercourse when having the symptoms h. Used condoms while having sexual intercourse during the time I had the symptoms i. Went to free services j. Did nothing | | | **Y N**  1 2  1 2  1 2  1 2  1 2  1 2  1 2  1 2  1 2  1 2 | ***b. Did it resolve the symptom?***  **Y N**  1 2  1 2  1 2  1 2  1 2  1 2  1 2  1 2  1 2  1 2 | |
| Q1104 | Have you ever been jaundiced? | | Yes 1  No 2  Don’t know 9 | | **🡪 Q1201**  **🡪 Q1201** | |
| Q1105 | When you were jaundiced, did you seek treatment? | | Yes 1  No 2  Don’t know 9 | |  | |

# SECTION 12: KNOWLEDGE, HIV RISK AND TESTING HISTORY

# I WILL ASK YOU ABOUT YOUR KNOWLEDGE AROUND HIV/AIDS.

| **No.** | **Questions** | **Coding of answers** | | | | **Skip to** | |
| --- | --- | --- | --- | --- | --- | --- | --- |
| Q1201 | Have you ever heard of HIV/AIDS before this interview? | Yes 1  No 2  No response 9 | | | | **→Q1301** | |
| Q1202 | In your opinion, can you tell if someone is infected with HIV just by looking at him/her? | Yes 1  No 2  Don’t know 9 | | | |  | |
| Q1203 | ***Now I am going to read some statements about HIV and AIDS. Some of them are true and some are not true. These are general statements and do not refer to your own experience or behavior. Please tell me whether you agree or disagree with each of the statements.*** | | | | | | |
|  | **Statement** | | **Response** | | | |  |
|  |  |  | **True** | **False** | **Don’t Know** | |  |
| A | Having sex with only one faithful partner who is not HIV infected reduces the risk of HIV transmission. | | 1 | 2 | 9 | |  |
| C | Using condom every time during vaginal sex can reduce HIV transmission. | | 1 | 2 | 9 | |  |
| C1 | A person who looks healthy can be infected with HIV | | 1 | 2 | 9 | |  |
| D | Mosquitoes and other insect bites will transmit HIV. | | 1 | 2 | 9 | |  |
| D1 | Eating with people living with HIV can get HIV transmission. | | 1 | 2 | 9 | |  |
| E | Sharing needles when injecting drugs will increase the risk of HIV infection. | | 1 | 2 | 9 | |  |
| F | Cleaning needles and syringes between injections reduces the risk of HIV. | | 1 | 2 | 9 | |  |
| H | Using condom every time during anal intercourse can reduce HIV transmission. | | 1 | 2 | 9 | |  |
| Q1204 | Could you please assess your risk for infection with HIV? | High risk 1  Low risk 2  No risk 3  Don’t know 9 | | | | | **→Q 1206**  **→Q 1207** |
| Q1205 | Why do you feel that you are at risk for HIV infection?  *(Do not read choices aloud. Just prompt for more responses and circle all that apply)* | **Yes No**  I often change sex partners 1 2  I don’t always use a condom 1 2  I use injected drugs 1 2  I’ve received blood transfusion 1 2  Other (specify) ……………………. 1 2 | | | | |  |
| **After complete 1205, skip to 1207** | | | | | | | |
| Q1206 | Why do you feel that you are not at risk for HIV infection?  *(Do not read choices aloud. Just prompt for more responses and circle all that apply)* | **Yes No**  I am faithful 1 2  I always use condoms 1 2  I have never injected drugs 1 2  I believe my partner is not infected 1 2  I don’t have anal sex 1 2  I never have sex with FSW 1 2  I’ve never received a blood transfusion 1 2  Other (specify) ……………………. 1 2 | | | |  | |
| Q1207 | Do you know of a place where you can get tested for HIV? | Yes 1  No 2  No response 9 | | | | **🡪Q 1209**  **🡪 Q1209** | |
| Q1208 | Have you ever had an HIV test? | Yes 1  No 2  Don’t know/No response 9 | | | | **🡪 Q1301**  **🡪 Q1301** | |
| Q1208a | If you do not mind sharing, and this will be kept confidential, what is your HIV status? | Positive 1  Negative 2  Did not receive results 3  Don’t know/No response 9 | | | | **🡪Q1209**  **🡪Q1209**  **🡪Q1209** | |
| Q1208b | If you had a positive result, have you registered in a clinic that specializes in HIV treatment? | Yes 1  No 2  Don’t know/No response 9 | | | |  | |
| Q1209 | When did you ***last*** take an HIV test for which you got the results? | In the past 6 months 1  In the past 12 months 2  Over one year ago 3  Never 4  Don’t know/no response 9 | | | | **🡪Q1215** | |
| Q1210 | The last time you received your HIV results, did you shared results with your sex partner, whether negative or positive? | Yes 1  No 2  Don’t know/No response 9 | | | |  | |
| Q1211 | That time, where did you take that test? | Public facilities (Preventive Medicine Center, VCT, Provincial/District Hospital, Provincial AIDS Center) 1  Private Center 2  06 Center 3  Mobile testing center 4  Other (specify)……………………………..5 | | | |  | |
| Q1212 | That time, did you pay for the test? | Yes 1  No 2  No response, Don’t know 9 | | | |  | |
| Q1213 | That time, who referred you to the testing center? | Community outreach worker 1  Healthcare worker 2  Sex partner 3  Injecting partner 4  Mass media 5  Other (specify)……………………………..6 | | | |  | |
| Q1214 | ***If Q1208=1,*** Have you ever disclosed your HIV status to any of your sex partners? | Yes 1  No 2  Don’t know/No response 9 | | | |  | |
| Q1215 | Have you encouraged your regular sex partners, either male or female, to get tested for HIV? | Yes 1  No 2  Don’t know/No response 9 | | | |  | |
| Q1216 | Have any of your regular sex partners, either male or female, ever told you they were HIV positive? | Yes 1  No 2  Don’t know/No response 9 | | | |  | |
| Q1217 | How often do you think you should get tested for HIV? | Every \|__\|__\| months  Others code 00  Don’t know 88  No response 99 | | | |  | |

**SECTION 13: PROVINCE SPECIFIC INTERVENTION ACCESS SECTION**

I WILL NOW ASK YOU ABOUT YOUR ACCESS TO HEALTH AND OTHER SERVICES.

| **No.** | **Questions** | **Coding of answers** | **Skip to** |
| --- | --- | --- | --- |
| Q1301 | When was the ***last time*** you received free clean syringes and needles? | In the last month 1  In the past 6 months 2  In the past 12 months 3  Over 1 year 4  Never 5  Don’t know/no response 9 | **→Q1304**  **→Q1304**  **→Q1304**  **→Q1304**  **→Q1304** |
| Q1302 | ***If in the last month***, how many times did you receive free clean needle/syringe? | [__\|__\|__] times  Don’t remember 999 |  |
| Q1303 | Who provided you the needles/syringes?  *(Do not read choices aloud. Just prompt for more responses and circle all that apply)* | Y N  Peer educators/Health educators 1 2  Healthcare workers 1 2  Fellow drug users 1 2  VCT center 1 2  Drop-in center 1 2  Sexual partners 1 2  Harm reduction box 1 2  Others (specify)___________ 1 2 |  |
| Q1304 | ***When was the last time,*** someone talked to you about safe injection? | In the last month 1  In the past 6 months 2  In the past 12 months 3  Over 1 year 4  Never 5  Don’t know/no response 9 | **→Q1307**  **→Q1307**  **→Q1307**  **→Q1307**  **→Q1307** |
| Q1305 | ***If yes***, how many times did you get this information ***in the last month?*** | [__\|__] times  Don’t remember 99 |  |
| Q1306 | Who have talked to you about safe injection?  *(Do not read choices aloud. Just prompt for more responses and circle all that apply)* | Y N  Peer educators/Health educators 1 2  Healthcare workers 1 2  Fellow drug users 1 2  VCT center workers 1 2  Drop-in center workers 1 2  Sexual partners 1 2  Others (specify)_______________ 1 2 |  |
| Q1307 | When was the ***last time*** you received free condoms? | In the last month 1  In the past 6 months 2  In the past 12 months 3  Over 1 year 4  Never 5  Don’t know/no response 9 | **→Q1310**  **→Q1310**  **→Q1310**  **→Q1310**  **→Q1310** |
| Q1308 | ***If yes***, how many times did you receive free condom ***in the last month?*** | [__\|__\|__] times  Don’t remember 999 |  |
| Q1309 | Who provided you the free condoms?  *(Do not read choices aloud. Just prompt for more responses and circle all that apply)* | Y N  Peer educators/Health educators 1 2  Healthcare workers 1 2  Fellow drug users 1 2  VCT center 1 2  Drop-in center 1 2  Sexual partners 1 2  Harm reduction boxes 1 2  Others (specify)___________ 1 2 |  |
| Q1310 | ***When was the last time*** someone talked to you about safe sex? | In the last month 1  In the past 6 months 2  In the past 12 months 3  Over 1 year 4  Never 5  Don’t know/no response 9 | **→Q1313**  **→Q1313**  **→Q1313**  **→Q1313**  **→Q1313** |
| Q1311 | ***If yes***, how many times did you get this information ***in the last months?*** | [__\|__] times  Don’t remember 99 |  |
| Q1312 | Who have talked to you about safe sex?    *(Do not read choices aloud. Just prompt for more responses and circle all that apply)* | Y N  Peer educators/Health educators 1 2  Health workers 1 2  Fellow drug users 1 2  VCT center workers 1 2  Drop-in center workers 1 2  Sexual partners 1 2  Others (specify)___________ 1 2 |  |
| Q1313 | ***When was the last time*** you received information or materials directed at people who inject drugs on safe sex? | In the last month 1  In the past 6 months 2  In the past 12 months 3  Over 1 year 4  Never 5  Don’t know/no response 9 | **→ Q1316**  **→ Q1316**  **→ Q1316**  **→ Q1316**  **→ Q1316** |
| Q1314 | ***If yes***, how many times did you receive this information ***in the last months?*** | [__\|__] times  Don’t remember 99 |  |
| Q1315 | How have you received the information?  *(Do not read choices aloud. Just prompt for more responses and circle all that apply)* | Y N  Peer educators/Health educators 1 2  Health workers 1 2  Fellow drug users 1 2  VCT center workers 1 2  Drop-in center workers 1 2  Sexual partners 1 2  Mass media 1 2  Internet 1 2  Cultural events 1 2  Other (specify)…………………….1 2 |  |
| Q1316 | ***In the last 12 months***, have you received the following support services?  *(Circle all that apply)* | Yes No  Treatment counseling 1 2  Occupational training 1 2  Job placement 1 2  Microfinance 1 2  Methadone treatment & counseling 1 2  Other services 1 2  Specify........................................................... |  |
| Q1317 | Do you know where you can get free care treatment services for people with HIV in the city where you’re living? | Yes 1  No 2 |  |
| Q1318 | ***If Q1208=1, ask***  Have you ever taken drugs for HIV treatment (ARV), even if only once?  *(Check yes even if have taken only once)* | Yes 1  Never before 2  Don’t remember/don’t know 9 |  |
| Q1319 | Do you know if home-based self-help group services are available for people living with HIV/AIDS? | Yes 1  No 2  No response 9 |  |
| Q1320 | Have you ever heard about Methadone treatment clinic? | Yes 1  No 2  No response 9 | **→ Q1323**  **→ Q1323** |
| Q1321 | If yes, have you accessed it? | Yes 1  No 2  No response 9 | **→ Q1323**  **→ Q1323** |
| Q1322 | If yes, are you currently receiving treatment from there? | Yes 1  No 2  No response 9 |  |
| Q1323 | In the past 6 months, have to visited any VCT sites, and in those 6 months, how many times? | No. of times \|___\|___\|  Don’t remember 99 |  |

***The Interview is completed here. Thank you very much for your help and time. Is there anything you want to discuss with us? Please go to the next room for counseling and testing procedure.***
